# What Is Senile-Pneumonia?

**Published:** 1869-08

**Authors:** 


### What is Senile-Pneumonia?

It is merely a ramollisment, or splenization in which the lungs are soaked as it were with dissolved blood, and so much softened as to be easily lacerable and reducible into a soft pap on pressure. Hence it is much more frequent in old and enfeebled subjects than in younger patients; it is frequently associated with disease of the left side of the heart.

There are two varieties of attack. It may be either sudden and manifest, setting in with a chill, pain in the side, cough, &c., or, it creeps on insidiously, and is well established before its existence is even suspected.

In March and April it is almost always manifest; often preceded for several days by headache, general languor, coryza with or without epistaxis; flying pains in the limbs and chest; when the Pneumonia is fairly established, stupor and adynamia quickly set in.

In the second or *latent* variety, there is no chill or pain; the patient only feels ill at ease and altogether more feeble; the breathing may be hurried and irregular, with some paroxysmal cough and difficult expectoration of phlegm. The skin may be merely warm, and tongue dry, without either cough or disturbance of breathing, although the patient may have been subject to asthma, or chronic cough. In short the only symptoms may be general malaise and feebleness.—*Ib.*

